# Secondary Prevention of Cardiovascular Diseases and Application of Technology for Early Diagnosis

**DOI:** 10.1155/2018/5767864

**Published:** 2018-05-08

**Authors:** Sachith Paramie Karunathilake, Gamage Upeksha Ganegoda

**Affiliations:** Faculty of Information Technology, University of Moratuwa, Katubedda, Moratuwa, Sri Lanka

## Abstract

Cardiovascular diseases result in millions of deaths around the globe annually, most of which are avoidable if identified early. Preventive healthcare has a major role in the fight against cardiovascular diseases. Primary, secondary, and tertiary prevention have their own applications along with benefits and drawbacks. This paper aims to elevate the sensitivity of “secondary prevention of cardiovascular diseases.” Firstly, it discusses common types of cardiovascular diseases around the globe and their causes. Secondly, it analyzes different risk factors associated with cardiovascular diseases and then discusses incoming technological trends in cardiovascular disease prediction and finally provides an insight into the importance of secondary prevention of cardiovascular diseases and commonly prescribed interventions for high risk patients.

## 1. Introduction

Health and wellbeing is one of the most primary and significant concerns for mankind. However this concern is constantly challenged by diseases and illnesses. While some of these diseases are fatal, some can be cured or their negative impacts could be minimized if diagnosed at early stages. The diseases that challenge the wellbeing of an organism can be categorized into two main categories based on the agent of the said disease. Diseases that are spread by infectious agents such as viruses and bacteria are referred to as communicable diseases while other diseases that are not caused by infectious diseases are known as noncommunicable diseases (NCD) that are caused by a combination of genetic, physiological, environmental, and behavioral factors. NCD result in an average fatality rate of 40 million lives annually which is 70% of global deaths [1]. Cardiovascular diseases cause an average of 17.7 million deaths each year (44% of NCD fatalities) making it one of the most deserving topics for research on prevention. Cardiovascular diseases (CVD) are a group of disorders of the heart and blood vessels which is the most significant cause of death globally. Despite the critical fatality rate 90% CVD can be prevented by taking necessary precautions [2].

CVD has both health and social impacts. Long term treatments for cardiovascular diseases demand significant financial resources. This could cause poverty in low and middle income families. Widespread of CVD may ultimately cause a burden on the economies of the country [3]. In countries where medical and healthcare sector is not advanced, diagnosis of CVD could be late, which would result in patient conditions irreversibly worsen or even death. This could reduce the life expectancy levels in the country.

There are three types of prevention mechanisms to prevent and reduce the impacts of a disease. Primary prevention refers to the steps taken by an individual to prevent the onset of the disease. This is achieved by maintaining a healthy lifestyle choice such as diet and exercise. Secondary prevention focuses on reducing the impact of the disease by early diagnosis prior to any critical and permanent damage. This facilitates avoiding life threatening situations and long term impairments from a disease. Tertiary prevention is used once long term effects set in, by helping the patients to manage pain, increase life expectancy, and increase the quality of life.

The secondary prevention of CVD includes diagnosis and prevention. Most critical step of secondary prevention is early diagnosis which allows medical professionals to provide required care for patients and improve the quality of life. This requires identifying risk factors, criticality of risk factors, and how the variation of these factors relates to CVD. Upon early diagnosis, patients could be directed to required treatments affording a higher quality of life.

Main attraction of secondary prevention over tertiary prevention comes from two factors. Factor one is the cost where the cost of secondary prevention is far less relative to tertiary prevention. Secondly it effects on the quality of life of the patient. Tertiary prevention involves major procedures that could cause discomfort to the patient as well as disrupt the daily activities, whereas secondary prevention focuses on less intense treatments which include drugs and lifestyle changes. Therefore creating awareness on secondary prevention could create positive impacts on individual lives as well as on a macroeconomic level.

## 2. Types of Common Cardiovascular Diseases

Cardiovascular diseases refer to all illnesses associated with heart and circulatory system. These illnesses are sometimes caused by modifiable risk factors such as diet, exercise, and other lifestyle choices while on certain occasions they are caused by unmodifiable factors such as age, gender, family history, and genetic predisposition for the disease [6]. These cardiovascular diseases have long lasting effects if not treated properly and are considered to be one of the most significant causes of death all around globe. Most common types of CVD include coronary artery diseases (CAD), cerebrovascular disease, peripheral arterial disease, and congenital heart disease. This section will provide an insight into common types of CVD's impacts and possible causes.

### 2.1. Coronary Artery Diseases

CAD, the most common type of CVD, refers to the condition where circulatory vessels that supply oxygenated blood to the heart get narrowed. This occurs due to a deposition of plaque (a combination of cholesterol, macrophage cells, calcium, and fibrous connective tissue) inside coronary arteries. This condition is referred to as atherosclerosis [7]. Once these plaques rupture, blood clots are formed inside the arteries which could lead to the partial or complete blockage of blood supply to the heart muscles. Symptoms of CAD include dyspnea (shortness of breath), myocardial infarction, and angina pectoris. Out of the above-mentioned symptoms, myocardial infarction and angina pectoris are frequently interchanged. Angina pectoris is a state in which the blood supply to the myocardium is significantly reduced thereby creating a squeezing or burning sensation at the sternum. However, myocardium necrosis has not yet occurred at this stage. In contrast, myocardial infarction which is commonly known as a heart attack is a state where, due to the unavailability of oxygenated blood, death of myocardial cells occurs. Both of these conditions can be identified using an electrocardiogram (ECG) where myocardial infarction presents with a ST segment (flat section of the ECG between the end of the S wave and the beginning of the T wave) depression or elevation and T wave inversion and angina pectoris present with only ST segment inversion. Research has identified several risk factors associated with CAD including cholesterol, smoking, obesity, and blood pressure [4, 8]. Apart from the above, diabetes mellitus (commonly referred to as diabetes) has a strong relationship with CAD. Studies have revealed that hyperglycemia accelerates the process of atherosclerosis by creating biochemical changes in the human body [9].

Out of those variables, research has identified that cholesterol and blood pressure contribute more towards CAD. When considering correlation of cholesterol and CAD, low density lipoprotein (LDL) cholesterol creates a higher risk in relation to HDL. In the case of blood pressure, it has been found out that stage 1 hypertension creates a higher risk for CAD [10].

### 2.2. Cerebrovascular Diseases

Cerebrovascular disease is a type of CVD associated with circulatory vessels that supply blood to the brain, causing the patient to have a stroke. The most common cause of cerebrovascular disease is hypertension which causes the artery inner lining to damage. This damage results in aggregation if there are platelets in the area where collagen is exposed. Four most common types of cerebrovascular diseases are stroke, transient ischemic attack (TIA), subarachnoid hemorrhage, and vascular dementia. Stroke occurs by a blockage of oxygenated blood to the brain due to thrombosis or embolism, which would lead to brain damage [11]. There exist three main types of causes for cardioembolic strokes, namely, arrhythmia, valve disorders, and cardiac chamber and wall abnormalities. Out of these causes atrial fibrillation (type of arrhythmia) is considered a major etiology of strokes [12]. Atrial fibrillation is a condition where the atrium fibrillates instead of fully contracting there by creating an irregular heartbeat. This fibrillation causes blood to pool allowing the formation of clots. These clots could block arteries that supply blood to the brain resulting in a stroke. TIA is a type of stroke that occurs temporarily with symptoms similar to a stroke. Subarachnoid hemorrhage is caused by blood leaking onto the surface of the brain or out of the arteries [13]. This leaked blood results in damaging brain tissue and neural structures.

Along with hypertension obesity, diabetes and smoking have been identified as the most leading causes for cerebrovascular diseases.

### 2.3. Congenital Heart Disease

Congenital heart diseases are associated with the structure of the heart. This condition is most commonly identified as birth defects, in the newborn children. Defects may vary such as structural defects of heart walls, heart valves, or even veins and arteries around the heart which could result in blocking blood flow, forcing the blood to flow in the wrong direction, and slowing down the blood flow. Symptoms of CHD are mostly identified at birth, but in certain cases patients may go undiagnosed for a long time or even their entire life. Common symptoms of CHD are heart murmur, underdeveloped limbs, and shortness of breath, fatigue, and cyanosis.

Causes for congenital heart diseases may not be directly identifiable. They could be caused by different factors such as infections during pregnancy (rubella), use of certain drugs, alcohol, and tobacco, genetic predisposition, or even poor nutrition. Treatment for CHD may depend on the severity of the defect. While in certain cases, treatment is not required, some might demand heart surgery in order to repair the defects or even heart transplants.

### 2.4. Peripheral Arterial Disease

A condition caused by reduced blood supply to limbs due to atherosclerosis (fatty deposits) in arteries is referred to as peripheral arterial disease (PAD). This is commonly associated with legs. Common symptoms of PAD include discoloration of legs, cramps in hip and calf muscles, and hair loss on limbs. However in many instances, these symptoms may go unnoticed. Most common risk factors of PAD include high blood pressure, smoking, diabetes, high blood lipids, and high levels of homocysteine. Out of these, smoking and diabetes have the biggest contribution to PAD as they reduce the blood flow to the limbs.

Peripheral arterial disease could lead to further complications such as critical limb ischemia where the open sores occur in limbs that are irrecoverable. These sores may cause tissue death in the limb which could ultimately lead to the amputation of the limb.

## 3. Risk Factors of Cardiovascular Diseases

Cardiovascular diseases may be caused as a result of many risk factors. These factors can be generally categorized into two groups, namely, modifiable risk factors and nonmodifiable risk factors. Modifiable risk factors refer to controllable causes of cardiovascular disease such as obesity, blood lipids, and behavioral factors. Nonmodifiable risk factors are those which cannot be controlled such as age, gender, and genetic predisposition. Awareness of these risk factors is highly critical in both stages of secondary prevention, early diagnosis, and treatment. Understanding risk factors and their interactions allow medical professionals to understand whether or not a particular individual is at risk and if so, how they could be controlled. This section provides an insight into few risk factors for CVD and their effect.

### 3.1. Gender

CVD is one of the most leading causes of death for people in both genders. However, statistical analysis shows that certain manifestations of CVD are more common in one gender relative to the other. It has been established that males are more prone to coronary heart diseases [14] while women have a higher risk of being subjected to strokes and heart failures [15]. A study conducted in Netherlands with 8419 participants has identified that the risk of CVD for men and women around the age of 55 is relatively similar. The estimated lifetime risk of CVD for men was 67.1% while women had a risk rate of 66.4% [16]. However, this research has found out that there are significant differences in the first manifestations of CVD in men and women. According to the research document, 27.2% and 22.8% of first manifestation for men were coronary heart disease and cerebrovascular heart diseases, respectively, while women showed rates of 16.9% and 29.8% for the above-mentioned CVD types, leading to the conclusion that men have a higher risk of coronary heart diseases, while women are exposed to a higher risk or cerebrovascular diseases.

**Figure 1:**
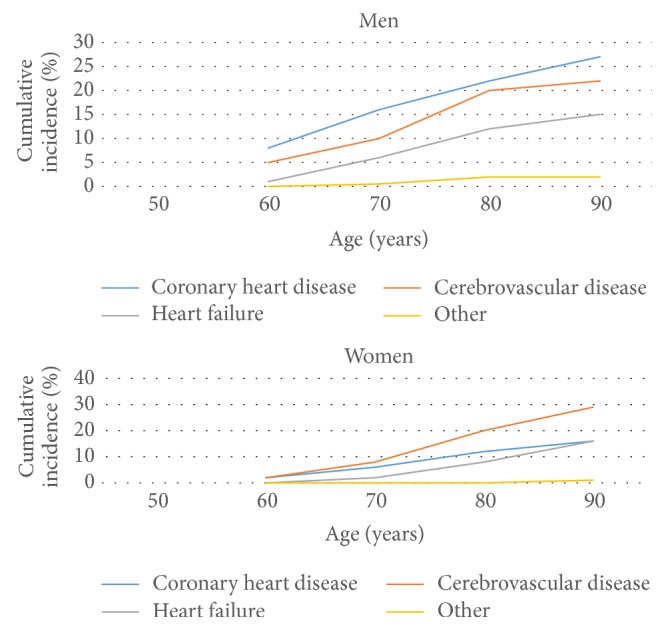
Correlation of age and CVD risk in men and women [4].

The general low susceptibility of women to cardiovascular diseases (as evident by Figure 1) can be attributed to cardioprotective effects of estrogen. Although the full effect of estrogen on women's cardiovascular health has not yet been identified, research shows that it contributes in increasing HDL cholesterol levels while decreasing LDL levels, which is crucial in preserving CV health. Furthermore, it is said that estrogen inhibits the development and progression of atherosclerosis. However, with menopause, due to reduction of estrogen, the susceptibility of women to CVD increases to approximately the same level as of men.

### 3.2. Age

Age is one of the most common nonmodifiable factors considered in almost all CVD risk prediction models. Age factor affects the two genders in a different manner for developing cardiovascular diseases. As mentioned in Figure 1, at a younger age, females have a less risk of developing CHD. However, this advantage reduces drastically over time. It has been found out that risk of CHD increases with age [10]. A reason for this would be the increase in the cholesterol levels with age. It has been estimated that total cholesterol of males increases till the age of 45–50 years while in females this period extends up to 60–65 years [17]. Furthermore increase in blood pressure with age could also be a cause for the increase in CHD risks. It is noteworthy to mention that increase of blood pressure is more prominent in women in relation to men [18]. However when considering multivariable risk assessment models, we can assume that age is an indicator of how long the person was exposed to other risk factors such as smoking and obesity creating a doubt whether or not age is an independent risk factor for CVD. It has been proven by an investigation that given the absence of glucose intolerance and moderate blood pressure and cholesterol levels, life expectancy can be extended up to 85 years [19]. This concludes that even though age is relevant risk factor in CVD, life expectancy can be increased with a modified lifestyle (it may still be affected by genetic factors).

### 3.3. Obesity

Obesity refers to the condition of accumulating of body fat leading to health risks. However association of obesity and CVD has been a long debated topic. While many studies show that obese individuals have a relative to higher risk to gain CVD, not many show a direct a correlation between weight/ obesity and CVD. Obesity is associated with many other risk factors such as lipids (cholesterol), glucose, and blood pressure which lead to the general consensus that risk of CVD for obese individuals is primarily due to the above said risk factors and not the obesity per se.

Body mass index (BMI) can be considered as a crude measurement of obesity. This is calculated by dividing the weight of an individual (Kg) by square of height (m2). BMI between 25 and 30 is considered overweight while a BMI above 30 is considered obese. However, association of BMI and CVD risk varies from individuals. As an example, in females, BMI less than 21 is considered to be great for protection from CVD. However, it has also been found out that even a BMI over 30 may not threat cardiovascular health as long as the fat is accumulated in the pelvis area and not the abdomen [20].

Causes for obesity could be either genetic or behavioral. While some are genetically programmed to retain fat, and lower metabolic rates, some may lead to unhealthy lifestyle with lack of exercise and unbalanced diets. American Heart Association (AHA) states that even a 5 to 10% decrease in body weight can have positive impacts such as decrease in blood pressure, cholesterol, and increased sensitivity to glucose, which would reduce the risk of CVD.

## 4. Technological Applications for Prediction of Cardiovascular Diseases

Advancement in technology has benefited mankind in many different ways. Application of technology in the field of medicine has enabled researchers and doctors to treat their patients more effectively and efficiently. With the recent advancements in artificial intelligence and data mining, medical personnel have the ability to extend their ability from treatment to early prediction of diseases. Early prediction allows patients to receive appropriate medical attention before the disease worsens leading to further complications such as myocardial infarctions, muscle death of limbs, or even death. Receiving treatment at an early stage not only increases the life expectancy of patients but also improves the quality of life. This section will focus on different technological techniques used for prediction of cardiovascular diseases and their effectiveness in a more technological perspective.

### 4.1. Data Mining

Data mining refers to the computational process analyzing large data sets and discovering patterns. In the context of medicine, data mining is processing large volumes of datasets created by medical professionals in order to uncover patterns which will aid in making patient related decisions [21].

**Figure 2:**
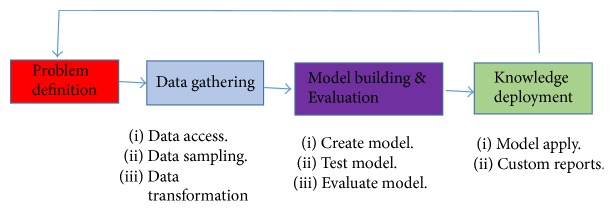
Data mining process [5].

This process is used mainly for two tasks, namely, descriptive and predictive tasks. Predictive tasks which are more applicable for disease prediction include uncovering hidden information and then extending these findings into the future in order to make predictions of future events using techniques such as artificial neural networks and machine learning. Some common techniques of predictive analysis include regression and classification. An abstract view of the data mining process is given in Figure 2.

#### 4.1.1. Associative Classification and Genetic Algorithm Method

Associative classification is a process which aims to identify relationships between variables. This allows researchers to create rules to interpret relationships between variables, which can solve classification problem uncertainty. Classifiers generate a wide range of rules using different approaches such as decision tree and Naïve Bayes. Later a small high quality subset of rules is selected using pruning techniques.

Akhil Jabbar et al. have used associative classification and genetic algorithms to implement a system to predict heart diseases. In order to improve the accuracy of the classifier, they have incorporated informative attribute entered rule generation and hypothesis testing *Z*-statistics, which has resulted in an accuracy rate of 89% for predicting heart diseases [22]. Some of the findings in their research are shown below.

Results of associative classification method are as follows [22]:

1. AGE > 45, BP Diastolic, BP systolic, diabetes => Heart Disease
2. AGE > 45, BP Diastolic, BP systolic, Hypertension, diabetes => Heart Disease
3. AGE > 45, BP Diastolic, diabetes => Heart Disease
4. AGE > 45, BP Diastolic, BP systolic, diabetes => Heart Disease
5. AGE > 45, BP Diastolic, Male, Hypertension, diabetes => Heart Disease
6. AGE > 45, BP Diastolic, Hypertension, diabetes => Heart Disease
7. AGE > 45, BP Diastolic, Hypertension, rural => Heart Disease
8. AGE > 45, BP systolic, Hypertension, diabetes => Heart Disease
9. AGE > 45, diabetes, rural => Heart Disease
10. AGE > 45, Male, Hypertension, diabetes => Heart Disease
11. AGE > 45, Male, Hypertension, rural => Heart Disease
12. AGE > 45, Hypertension, diabetes => Heart Disease

*Results*.

1. Majority of the people who had CVD were in the age group of 46–65.
2. Among all the participants of the study 60% of the males and 40% of the females had heart disease.
3. 50% of the males who had hypertension are associated with CVD.
4. 8% of the females who had hypertension are associated with CVD.
5. A higher percentage of males were found to be diabetic.
6. 38% of the people who live in urban areas are associated with heart disease.
7. Hypertension and diabetes account for 30% of all cases.
8. Among all the cases males had a higher systolic pressure (44% cases).
9. 32% of males who live in urban areas are associated more with heart disease.

#### 4.1.2. Prediction Using Classification Analysis and Regression Trees

A research has been conducted to predict heart diseases by classifying phonocardiograms (a record of sounds and murmurs made by the heart) using regression trees and classification analysis. This aims to identify pathological murmurs in order to predict the onset of disease process by involving three steps as follows [23].

1. Extracting and processing PCG signals in order to isolate heart sounds by removing noise.
2. Extracting features from the signals which are more critical in the classification process.
3. Generating a decision tree by splitting intermediate nodes into two child nodes with the objective of increasing the homogeneity of terminal nodes.

#### 4.1.3. Disease Prediction Using Naïve Bayes and Laplace Smoothing

Another research associated with data mining where the researchers implemented a system to predict heart diseases using Naïve Bayes algorithm, which is used to create models that have predictive capabilities which have high dimensional inputs.

This research focuses on 13 inputs in order to predict CVD: age, gender, chest pain type, fasting blood sugar, ECG, exercise induced angina, slope, CA, thallium test, blood pressure, old peak, maximum heart rate achieved, and serum cholesterol. However, they have also implemented a mechanism to use 6 of the above inputs in order to arrive at predictions. The accuracy rates of two mechanisms are significantly different where 6 inputs generated an accuracy rate of 62% while 13 inputs generated an 86% accuracy [24].

#### 4.1.4. Heart Disease Prediction System (HDPS) [25]

**Figure 3:**
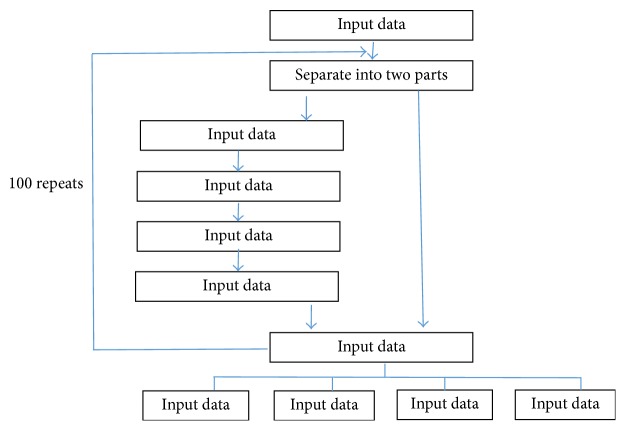
HDPS process overview.

A research conducted in Taiwan has produced a mechanism using an Artificial Neural Network (ANN) for classification and 14 attributes as follows: gender, chest pain type, resting blood pressure, serum cholesterol, fasting blood sugar, ECG, maximum heart rate achieved, exercise induced angina, old peak, slope, number of major blood vessels colored by fluoroscopy, and thal. The proposed network is a three-layer model with an input layer, hidden layer, and output layer. Each layer consists of 13, 6, and 2 neurons, respectively. Each attribute is assigned with random weights at the beginning and are later revised during the training process in order to match the testing data set (see Figure 3).

This research has yielded an 80% accuracy rate in predicting heart diseases.

## 5. Secondary Prevention Intervention for CVD and Its Importance

Secondary prevention aims to identify a disease within a patient before the onset of symptoms and reduce the impact on the life of the patients. While knowledge on the variation of risk factors aids in the screening process, it is important to have an understanding of medical interventions necessary to reduce the impact of the disease. This section focuses on lifestyle interventions and medications associated with secondary prevention of cardiovascular diseases.

### 5.1. Quality of Life Improvement

In cases where primary prevention fails due to unmodifiable risk factors, secondary prevention becomes the next best choice in maintaining the quality of life of the patient. Secondary prevention comprises identifying risks of CVD before it does permanent damage or create critical medical situations and then conducting necessary interventions to reverse the effects of the disease. These treatments have a relatively low impact on the patient relative to tertiary interventions. If a patient is diagnosed to have a risk of CVD, using the techniques discussed in the previous section or any other traditional methods, then he/she is prescribed for two types of interventions. First type of intervention is lifestyle changes, which does a minimum impact on an individual. Secondly, medical interventions are far more affordable than most tertiary interventions. Certain tertiary procedures such as pacemaker implementation require constant care throughout the patient life. For instance, avoiding prolonged exposure to electromagnetic fields and regular visits to medical professionals could disrupt certain jobs or even regular life of the patient.

Higher affordability of secondary prevention interventions ensures the usage of such treatments to ensure the patient safety. In critical unaffordable medical procedures (tertiary prevention), there are instances where patients procrastinate due to financial difficulties putting their health in grave danger. Another common occurrence is patients visiting developing countries to receive affordable care. Secondary prevention methods could minimize this type of inconveniences to patients.

Thirdly, secondary prevention reduces the socioeconomic burden on the nation as well as to individual households. Considering household burden, it comes in short term as well as long term. Short term costs include hospitalizations costs, ambulance rides, and surgery expenditure. Long term costs include doctors' appointments, tests to monitor disease progression, and medicine. This effect may be multiplied due to the lack of productivity of patients or if patient dies. Lack of productivity also has macroeconomic level implications. It has been estimated that, in 2010, there has been a loss of $41.7 billion in productivity due to CVD related employee morbidity and $137.4 billion due to premature death.

### 5.2. Medical Interventions

The most common cause of CVD is dyslipidemia (abnormal amounts of lipids in the blood). This leads to atherosclerotic CVD. Statin therapy is commonly used in order to manage blood lipids by medical professionals. This is a lipid lowering drug type/drug class that inhibits the body from creating cholesterol. Statins are often used in primary prevention as well as in secondary prevention. Studies show that these drugs lower the mortality rate by 15–20% while lowering nonfatal cardiovascular events at an even greater degree.

Another common cause of CVD is hypertension which is treated by beta blockers. These drugs reduce the effect of adrenaline, thereby lowering the heart rate of the patient. These are commonly prescribed for angina, myocardial infarctions, and arrhythmia. Target blood pressure for CVD risk individuals is <140/90 mmHg.

Most significant benefit of these medical interventions is that they are far more affordable compared to major procedures such as bypass surgery and stent replacement in tertiary prevention. Average price of beta blockers varies around $10–50 and of statin treatments drugs $10–20, thereby relieving the financial burden on the patient as well as on the economy of the country.

### 5.3. Lifestyle Interventions

Nonmedical interventions for CVD are mainly comprised of behavior modifications of high risk individuals for CVD. The following are some behavioral modifications to reduce the onset of CVD types.

Weight reduction is one of the most discussed lifestyle interventions under this topic. It is advised to maintain an average weight with a BMI between 18.5 and 24.9. It is also advised to maintain a waist line <35 for women and <40 for men. Dietary sodium reduction has also been prescribed to minimize CVD risk in individual. Sodium (consumed as salt) causes water retention which then leads to higher blood pressure. As discussed in previous chapters, high blood pressure may lead to different types of CVD as well as kidney damage. Other lifestyle modifications include complete cessation of smoking, physical activity (30–60 minutes of daily aerobic activity), and stress maintenance.

Dietary restrictions differ on the risk factor of CVD. Adults who are risked due to blood lipids are recommended to fruits, vegetables, whole grains, poultry, fish, and low fat dairy products while restricting sugar sweetened beverages, sweets, and red meat. It is also recommended to reduce the calories from saturated fat to 5-6% of daily calorie intake. Adults who have a higher BP are advised to follow the same dietary restrictions with lower sodium levels as discussed above.

Light to moderate alcohol consumption is associated with protection from CVD. Research shows that an alcohol consumption of 26 g/d provides maximal protection against CVD mortality.

## 6. Discussion

Cardiovascular diseases are a type of noncommunicable disease that has the highest fatality rate recorded. It has been calculated that approximately 17.7 million lives are lost to CVD each year globally. Furthermore, estimates show that, by year 2020, CVD will surpass infectious diseases and become the world's leading cause of death. Even if the disease does not cause death, conditions such as myocardial infarctions can create long term impacts on the patient which may reduce the quality of life and reduce the lifespan of the patient. For instance, an acute myocardial infarction can kill approximately one billion myocardial cells which cannot be regenerated. As a result of the damage, myocardium loses its ability to function synchronously leading to irregular heartbeats known as arrhythmia which requires long term medication or even implantable cardioverter defibrillators (ICD) if not resolved by medication. Such criticality demands the attention of preventive healthcare in all its formats.

Preventive healthcare has three main stages. Primary prevention of CVD refers to the adjusting modifiable risk factors in order to prevent the onset of disease. This includes lifestyle changes such as diets and regular exercise, discarding harmful behaviors such as smoking. Constant monitoring of risk factors would allow the patients to keep them in check thus leading to a healthier cardiovascular system. However, the one drawback of primary prevention is that it only focuses on modifiable risk factors. Although lifestyles play a major factor to susceptibility of CVD, there are many other genetic and environmental factors that cannot be controlled by an individual. This drawback of primary prevention is addressed by secondary prevention which focuses on early detection of diseases prior to critical and permanent damage, allowing the medical professionals to treat the patients and secure the quality of life. Early detection requires an in-depth knowledge of the disease itself, family history of the patient, lifestyle, and many other related factors. Although the process is more complex compared to primary prevention, benefits are far superior to tertiary prevention in terms of quality of life of patients as well as in a financial perspective. Medical professionals ordinarily use indicators such as age, blood sugar levels, and lipids to predict CVD and screen for particular diseases. However with the involvement of technology in the field of medicine, secondary prevention has evolved into a state where potential for CVD can be identified with accuracy rates approximating 80%. This is done by analyzing CVD trends in mass populations using data mining techniques and then applying the conclusions and trends to individuals to find their susceptibility to the disease.

Most prominent advantage of using technology for the prediction process is the accuracy and efficiency of the process. Traditional risk scores usually weight a predefined set of variables and then estimate a risk score for an individual. Studies show that traditional risk calculators tend to overestimate CVD risk in individuals where new AHA-ACC-ASCVD tool overestimated the risk by 86% and the ATPIII-FRS-CHD overestimated CVD occurrences 2.5 times [25]. Such inaccuracies may cause medical practitioners and the public to lose trust in disease prediction which could eventually lead to neglecting risk factors. However, technique such as ANN has the capacity to identify correlations between different risk factors. This could lead to identifying previously unknown correlations and rules for risk prediction which in return could improve the accuracy of assessment. Accurate risk identification allows medical professionals to intervene in managing risk factors using treatment and behavioral modifications prior to onset of more critical conditions, thereby improving the quality of life of the patient. Although accurate, there exists a drawback in technological risk prediction systems as well as in traditional risk scores. This drawback is the inability to predict the disease type (CAD, CHF, and PAD), which would allow more precise and focused treatment for the patients. This drawback could be addressed in the future by attributing disease type in the classification process, thereby predicting not only the probability of disease, but also the disease type.

Upon diagnosis, patients are directed to appropriate treatments. As an example, beta blockers and calcium channel blockers for blood pressure lowering could reduce the risk of cerebrovascular disease and coronary heart disease. Furthermore statin treatment to lower blood lipids could reduce the risk of atherosclerosis which is the cause for many cardiovascular diseases. The most notable fact is that treatments associated with secondary prevention can be carried out with the minimal effect to the daily life and the quality of the life, unlike tertiary prevention.

Tertiary prevention refers to intensive procedures conducted in order to increase life expectancy and manage pain. Some common procedures include bypass surgery, coronary angioplasty, defibrillators, stents, and pacemakers. In 2006, United States itself has conducted 418000 pacemaker procedures, 114000 defibrillator procedures, and 448000 bypass surgeries. Cost of these procedures may not be affordable to middle income individuals as opposed to secondary prevention treatments. A bypass surgery may cost an average of $75,000 and a stent insertion may have a cost run of $28,000. As a result, it is a common occurrence that patients in developed countries visit low income countries such as India and Thailand for such procedures.

Another notable drawback of tertiary prevention in relation to secondary prevention is the disruption of daily activities of the patient. A traditional bypass surgery may take approximately 8–12 weeks of recovery period and ongoing care after surgery with EKS, stress testing, and CT scans. Pacemakers require patients to be extra careful of their surroundings in order to avoid prolonged exposure to electromagnetic fields. Furthermore, regular follow-up with a medical professional is required to assure the correct operation of the pacemaker. Such difficult situations may be avoided by secondary prevention.

However, there are limitations associated with secondary prevention as well. Secondary prevention requires early discovery of diseases. But multiple risk factors act differently in individuals. Therefore, there is no correct way to precisely determine the onset of disease. Ongoing research focuses on creating prediction algorithms which can produce higher accuracy rates in disease prediction. Another barrier to secondary prevention is lack of resources. Although this may not be a problem in developed countries, developing and underdeveloped countries lack resources to screen large number of individuals for CVD. Unavailability of technological equipment and knowledge required for screening and lack of financial support may get in the way of secondary prevention.

A brief comparison of primary, secondary, and tertiary prevention methods is given in Table 1.

The comparison in Table 1 leads to the conclusion that primary prevention has the minimum burden on an individual. But as not every risk factor can be mitigated (unmodifiable risk factors), we have to consider the possibility that there may be instances where CVD is unpreventable. In those scenarios, secondary prevention can be considered the best course of treatment as it diagnoses the disease prior to any permanent damage and then reduces critical risk with minimum impact to patient life and financial status.

## 7. Conclusion

Cardiovascular disease is a noncommunicable disease with one of the highest fatality rates. Approximately 44% of total NCD deaths are caused by CVD. Even if the patient survived, long term treatments and procedures once the symptoms are set could be unaffordable to middle class individuals which in the long run would create a socioeconomic burden at the national scale. Due to this criticality of CVD, there exists a demand for procedures to reduce the negative impact of CVD. Secondary prevention plays a vital role in the said task, as it aims to identify diseases at early stages and then treat them prior to any critical damage.

Preventive healthcare is comprised of three main platforms. First, there is primary prevention, which suggests that patients should live in a way that h/she would not be a victim of the disease in the first place. In relation to CVD, this means maintaining ideal bodyweight, balanced diets, and cessation from unhealthy practices such as smoking and excessive alcohol consumption. However, CVD is a result of many factors which are modifiable as well as unmodifiable risk factors. Tertiary prevention aims to treat patients when the symptoms have been set and critical damage has already occurred. This in general aims to increase the life expectancy and quality of life of the patient via intensive procedures such as pacemaker placement and bypass surgery.

Early diagnosis plays a crucial role in secondary prevention. This requires intensive knowledge of risk factors contributing to CVD and different interactions among them. Some common risk factors of CVD include obesity, gender, age, blood lipids, and smoking. Since the invention of artificial intelligence CVD prediction has evolved into a new level, where machines are able to analyze millions of data sets and identify relations between different risk factors. These systems include either statistical model or artificial neural networks where some showed an accuracy rate over 85%. Average accuracy rates of these systems lie within 70–80%. This technological application has enabled medical professionals to diagnose high risk individuals of CVD, who are then treated prior to any critical condition such as myocardial infarction. This is advantageous as once such situation occurs, damage that occurs may be irrecoverable which may cause long term complications. For instance, cell death in heart may cause remaining cells in the heart to deform (enlarge), which could cause arrhythmia in the long run. Another benefit of secondary prevention is the significant cost savings it has over tertiary prevention. Tertiary prevention takes place when permanent damage or critical conditions occur. They focus on extending patient life and quality. However, constant care must be given after tertiary prevention treatments where it takes a prolonged time for the patient to adjust to daily activities. Furthermore, these said procedures may have financial costs which are unaffordable for middle income families. Upon early stage diagnosis, patients are prescribed with drug interventions as well as lifestyle interventions to reduce the risk of CVD. Drugs such as beta blockers and statin therapy could reduce risk factors of patients thereby relieving their risk of severe CVD diseases with long term effects. Lifestyle interventions include weight loss, cessation of smoking, limiting alcohol usage, and dietary restrictions. These interventions have a minimum effect on the patient's quality of life as well as economic status.

In conclusion, secondary prevention plays a vital role in the world's fight against CVD. It is highly probable with the advancement of medicine and technology; humankind will be able to predict CVD far more accurate than it is today, thereby improving the lives of millions around the world.

## Figures and Tables

**Table 1:** Comparison of primary, secondary, and tertiary prevention methods.

Aspect	Primary prevention	Secondary prevention	Tertiary prevention
Focus	Preventing the onset of disease, by eliminating risk factors	Reducing the impact of the disease by early diagnosis before permanent damage	Managing pain and increasing life expectancy of the patient

Financial burden	Very low: mainly focusing on lifestyle changes	Medium: medical interventions cost affordable amounts	Very high: surgery and other major procedures

Disruption of daily work	Low	Medium	High: requiring constant attention after medical interventions

Examples	Daily exercise, healthy diets	Beta blockers, statin treatment, lifestyle changes	Stent replacement, bypass surgery

## References

[1] Noncommunicable diseases. http://www.who.int/mediacentre/factsheets/fs355/en.

[2] McGill H. C., McMahan C. A., Gidding S. S. (2008). Preventing heart disease in the 21st century implications of the pathobiological determinants of atherosclerosis in youth (PDAY) study.

[3] Cardiovascular diseases (CVDs). http://www.who.int/mediacentre/factsheets/fs317/en/.

[4] Kannel W. B., McGee D. L. (1979). Diabetes and glucose tolerance as risk factors for cardiovascular disease: the Framingham study.

[5] Oracle https://docs.oracle.com/cd/B28359_01/datamine.111/b28129/process.htm#DMCON046.

[6] Scheuner M. T. (2001). Genetic predisposition to coronary artery disease.

[7] Coronary Heart Disease. https://www.nhlbi.nih.gov/health/health-topics/topics/cad.

[8] Gordon T., Castelli W. P., Hjortland M. C., Kannel W. B., Dawber T. R. (1977). Diabetes, blood lipids, and the role of obesity in coronary heart disease risk for women. The Framingham study.

[9] Stephanie Cooper J. H. C. (1999). Coronary Artery Disease in People With Diabetes: Diagnostic and Risk Factor Evaluation.

[10] Jousilahti P., Vartiainen E., Tuomilehto J., Puska P. (1999). Sex, age, cardiovascular risk factors, and coronary heart disease: a prospective follow-up study of 14 786 middle-aged men and women in Finland.

[11] https://www.nlm.nih.gov/.

[12] Maier I. L., Schregel K., Karch A. (2017). Association between Embolic Stroke Patterns, ESUS Etiology, and New Diagnosis of Atrial Fibrillation: A Secondary Data Analysis of the Find-AF Trial.

[13] Cerebrovascular disease - Introduction - NHS Choices. https://www.nhs.uk.

[14] Lloyd-Jones D. M., Larson M. G., Beiser A., Levy D. (1999). Lifetime risk of developing coronary heart disease.

[15] Lloyd-Jones D. M., Larson M. G., Leip E. P. (2002). Lifetime risk for developing congestive heart failure: the Framingham Heart study.

[16] Leening M. J. G., Ferket B. S., Steyerberg E. W. (2014). Sex differences in lifetime risk and first manifestation of cardiovascular disease: Prospective population based cohort study.

[17] Jousilahti P., Vartiainen E., Tuomilehto J., Puska P. (1996). Twenty-Year Dynamics of Serum Cholesterol Levels in the Middle-Aged Population of Eastern Finland.

[18] (1993).

[19] Terry D. F., Pencina M. J., Vasan R. S. (2005). Cardiovascular risk factors predictive for survival and morbidity-free survival in the oldest-old Framingham Heart Study participants.

[20] Eckel R. H., Krauss R. M. (1998). American Heart Association call to action: obesity as a major risk factor for coronary heart disease.

[21] Subanya B., Rajalaxmi R. R. Feature selection using artificial bee colony for cardiovascular disease classification.

[22] Akhil Jabbar M., Deekshatulu B. L., Chandra P. M.Akhil jabbar, Heart Disease Prediction System using Associative Classification and Genetic Algorithm.

[23] Amiri A. M., Armano G. Early diagnosis of heart disease using classification and regression trees.

[24] Vincy Cherian B. M. (2017). Heart Disease Prediction Using Na*∩*ve Bayes Algorithm and Laplace Smoothing Technique.

[25] DeFilippis A. P. (2015). Analysis of Calibration and Discrimination Among Multiple Cardiovascular Risk Scores in a Modern Multiethnic Cohort.

